# Adolescent Perceptions of Parenting Styles in Sweden, Italy and Greece: An Exploratory Study

**DOI:** 10.5964/ejop.v11i2.887

**Published:** 2015-05-29

**Authors:** Maria Giulia Olivari, Elisabeth Hertfelt Wahn, Katerina Maridaki-Kassotaki, Katerina Antonopoulou, Emanuela Confalonieri

**Affiliations:** a Psychology Department, CRIdee, Università Cattolica del Sacro Cuore, Milano, Italy; b School of Life Sciences, University of Skövde, Skövde, Sweden; c Department of Home Economics and Ecology, Harokopio University of Athens, Athens, Greece; Aalborg University, Aalborg, Denmark

**Keywords:** adolescents, parenting styles, PSDQ, country comparison, culture

## Abstract

Comparative research on parenting styles among Nordic and Mediterranean countries is still missing, despite the increasing number of studies on parenting styles in adolescence. This study explores similarities and differences in adolescents’ retrospective perceptions of parenting styles, for both parents, in Sweden, Italy and Greece, using the Parenting Styles and Dimensions Questionnaire. In particular, it examines the relation between parental role, adolescent gender, country of origin, SES and these perceptions. Swedish, Italian and Greek adolescents (N = 702; 30.9% Swedish, 39.6% Italian and 29.5% Greek) participated in the study. To test the principal effects three mixed 2(parent; mother and father)*2(gender; girl and boy)*3(countries; Sweden, Italy and Greece)*3(SES; low, medium and high) ANOVAs were conducted separately for each parenting style. To verify the interaction effects, a mixed 2(parent; mother and father)*3(countries; Sweden, Italy and Greece)*3(SES; low, medium and high) ANOVA was tested on authoritative style. Regarding authoritarian and permissive two mixed 2(parent; mother and father)*2(gender; girl and boy)*3(countries; Sweden, Italy and Greece) ANOVAs were tested. Mothers, as compared to fathers, were perceived as more authoritative, authoritarian and permissive. Moreover, boys perceived their parents as more authoritarian and more permissive than girls. Swedish parents were perceived as significantly less authoritarian than Italian and Greek parents and more permissive than Italian parents; Greek parents were perceived as less authoritarian and more permissive than Italian parents. The study provides an interesting contribution to parenting styles literature, showing how country legislation concerning family matters and SES are related the perception of parenting behaviours.

According to Darling and Steinberg (1993), parenting styles are a “constellation of attitudes toward the child that are communicated to the child and create an emotional climate in which the parents’ behaviours are expressed” (p. 493). In the literature, the most commonly investigated parenting styles are the authoritative, the authoritarian and the permissive styles (Baumrind, 1991; Moilanen, Rasmussen, & Padilla-Walker, 2014; Olivari, Tagliabue, & Confalonieri, 2013). Authoritative parents are demanding and responsive. They enhance children’s involvement and participation in family life through democratic behaviours, trusting and supporting their children, and controlling them without being restrictive. Authoritarian parents are highly demanding and directive, but not responsive. They exercise high control of their children and educate them through strict and punitive discipline measures. Permissive parents are highly responsive, but not demanding. They do not control their children and set few rules, warmly accepting their children and their behaviours. Mothers and fathers shape their preferable parenting style by being or not responsive and demanding towards their child (Confalonieri et al., 2010). Parenting style research has often focused on the study of an overall parenting style, assuming that both fathers and mothers adopt the same style or interdependent styles, or it has concentrated on the mother’s parenting style, overlooking the role of the father (Ge, Best, Conger, & Simons, 1996; Parke, 2000; Rohner & Britner, 2002; Sentse, Veenstra, Lindenberg, Verhulst, & Ormel, 2009).

According to Belsky’s model on the determinants of parenting (Belsky, 1984) and Bronfenbrenner’s ecological system theory (Bronfenbrenner, 1979, 1986), the social context plays an important role that can either support or thwart parental practice and behaviours. Researchers have long argued that parenting styles are affected by socio-economic status (SES), and several studies confirm this hypothesis (Hoff, Laursen, & Tardif, 2002). Bronfenbrenner (1958) highlighted that in middle-class families parent-child relationships were more acceptant and equalitarian, while in working-class families they were oriented towards discipline and obedience. Chen, Dong, and Zhou (1997) highlighted that parental occupation and education correlated positively with authoritative parenting for both parents, while they correlated negatively with authoritarian parenting for mothers. Additional evidence derives from von der Lippe’s (1999) study, which shows that less-educated mothers reported less authoritative parenting compared to more-educated mothers. More recently, Worden and Carlson (2005), as well as Gracia and Herrero (2008), suggested that the higher-educated parents are more likely to reject the acceptability of physical punishment of children.

Besides the role played by SES, there is strong empirical evidence suggesting that parents from different cultures and countries can adopt some common and some different culture-specific child-rearing values and goals, according to which they develop appropriate parenting styles (Bornstein, 1995; Harkness, Super, & van Tijen, 2000; Harwood, Handwerker, Schoelmerich, & Leyendecker, 2001). Within different countries, country legislation concerning family matters, as one element of the social context, may indirectly affect parental behaviours (Garbarino, Vorrasi, & Kostelny, 2002). Despite the growing interest in the investigation of parenting styles during childhood and adolescence (Olivari et al., 2013), few studies have examined the above issue among European countries (Ciairano, Kliewer, Bonino, & Bosma, 2008), whereas there are no comparative data for Nordic and Mediterranean countries. In particular, Sweden, Italy and Greece have different legislation and policies concerning the role of the family in the education and care of children, and these elements in policy-making may affect the family climate and family responsibilities. Table 1 summarises the information regarding the legislation, the cultural norms and values related to parenting, and the main research results on parenting styles for each of these three countries.

**Table 1:** Legislation, Cultural Norms and Values Related to Parenting, and the Main Research Results on Parenting Styles in Sweden, Italy and Greece

Country	Legislation	Cultural norms and values related to parenting	Research on parenting styles
Sweden	In 1979, it was the first country in the world to pass legislation prohibiting the use of physical punishment and other forms of insulting treatment toward children (Durrant, 2003). Corporal punishment was explicitly prohibited in an amendment to the Parenthood and Guardianship Code which states (art. 6.1): *“Children are entitled to care, security and a good upbringing. Children are to be treated with respect for their person and individuality and may not be subjected to corporal punishment or any other humiliating treatment.”*	The political sphere directs a great deal of resources towards ensuring gender equality in family, where mothers and fathers share equal responsibilities and opportunities in their children’s education (Allard, Haas, & Hwang, 2007; Harkness et al., 2001). The Swedish legislation has contributed to the formation of a negative attitude toward physical punishment in Sweden (Deley, 1988; Durrant, Broberg, & Rose-Krasnor, 1999). According to Sorbring and Gurdal (2011), Swedish parents hold more progressive, in terms of valuing autonomy, than authoritarian attitudes. They emphasize the children’s rights to express themselves and their opinions; they see the children as individuals with their own rights and own potential and support them in their skills and potential.	A recent study (Trifan, Stattin, & Tilton-Weaver, 2014) examined changes in authoritarian Swedish parenting practices across 50 years. The findings suggest that authoritarian parenting practices have declined strongly and that family environments have moved toward a more egalitarian pattern. Moreover, research on parenting practices has shown a decrease in the use of punitive parenting practices (Durrant, Rose-Krasnor, & Broberg, 2003; Straus, 2010).
Italy	In 1996, a Supreme Court judgment outlawed all violence in child-rearing (Judge Ippolito, Supreme Court of Cassation, 18 March 1996). Article 571 of the Criminal Code of 1975 states: “*Whoever misuses means of correction or discipline to harm a person subject to his authority, or entrusted to him for purposes of education, instruction, treatment, supervision or custody … shall be punished.*” The offence of abuse of correctional methods is applicable if there is a relationship of authority between the abuser and the abused, if the abuse results in physical or mental injury, and if it involves illegitimate correctional methods. Since then, corporal punishment is no longer a legitimate method of discipline. However, there has been no law reform to confirm the judgment in legislation by amending/repealing Article 571 or enacting explicit prohibition of corporal punishment in the home, though a number of bills have been proposed over the years.	During the last 20 years, a passage from the “ethical family” to the “affective family” occurred among Italian culture. In the “ethical family,” there were strict rules and boundaries among the generations. The parents’ roles were different: the mother was devoted to the care of children and the father was focused on providing rules and transmitting values (Confalonieri et al., 2010). Nowadays, in the affective family both mothers and fathers identify their main function as ensuring the care for the children, no more providing rules of conduct and focusing only on the transmission of love, to raise “happy” children (Pietropolli Charmet, 1995, 2000; Scabini & Cigoli, 2000). This parental change seems to involve more the fathers (Bacchini, Galiani, Guerriera, & Sbandi, 2003) who seem to have left their ethical tasks, moving on to the affective tasks that traditionally belong to the maternal figure.	A recent study (Confalonieri et al., 2010) examined and compared adolescents coming from northern and southern areas as to their perceptions about paternal and maternal parenting styles. The study found that Italian adolescents perceive their parents as being more authoritative than authoritarian and permissive. Considering the adolescent gender, it found that boys perceived their parents as more authoritarian and permissive than girls.
Greece	In 2006, legislation against corporal punishment within the family context was approved. Article 4 of Law 3500/2006 on the Combating of Intra-family Violence (in force 2007) states: “*Physical violence against children as a disciplinary measure in the context of their upbringing brings the consequences of Article 1532 of the Civil Code.*” Article 1532 of the Civil Code addresses abuse of parental authority.	The country has been described as a collectivist society (Hofstede, 1980) in which persons are considered integral members of larger social networks (Georgas, 1993; Georgas, Berry, Shaw, Christakopoulou, & Mylonas, 1996; Greenfield & Suzuki, 1998). According to Zervides and Knowles (2007), traditional Greek culture enhances family loyalty, adherence to group norms and maintenance of harmony in relationships with group members. It has been suggested that this type of value system is linked to severe and controlling child-rearing practices (Rosenthal, 1984), underlying the importance of conformity and obedience to parental rules (Papps, Walker, Trimboli, & Trimboli, 1995; Sprott, 1994; Szapocznik & Kurtines, 1993; Triandis, 1989).	Greek parents were categorized into four types: authoritative, authoritarian, permissive and strict (Antonopoulou & Tsitsas, 2011; Maridaki-Kassotaki, 2009). The strict style brings together features from the authoritarian and the authoritative styles. Strict parents are the parents who criticize, scold and punish their children as authoritarian parents do. They, however, explain to their children why they punish them and try to improve their behavior. They emphasize to their children that it is important to follow the rules of the family. The above behavioral features are lacking in the behavior of the authoritarian parents. A recent study (Antonopoulou, Alexopoulos, & Maridaki-Kassotaki, 2012) investigated the role of Greek father’s in the psychosocial development of children, showing that the children who perceived their fathers as authoritative had higher levels of empathy and self-esteem compared to children who perceived their fathers as being authoritarian.

Therefore, the purpose of the present study was to explore similarities and differences in adolescents’ retrospective perceptions of authoritative, authoritarian and permissive parenting styles for fathers and mothers in Sweden, Italy and Greece, considering the potential role played by the country legislation on family matters in affecting parenting styles across the three different countries. In particular, it sought to examine the relation between parental role, adolescent gender, country of origin, SES and these perceptions.

## Method

### Participants

Participants were 805 adolescents (279 Swedish, 301 Italian and 225 Greek) who filled in a questionnaire about their parents’ parenting behaviours during their childhood. Data from 702 adolescents (87.20% of the sample; 46.4% males and 53.6% females; 30.9% Swedish, 39.6% Italian and 29.5% Greek) who are natives in the country and answered at least 60% of the items regarding both their fathers and mothers are considered valid for the present study. Missing data on single items were computed through EM analysis (Dempster, Laird, & Rubin, 1977). Adolescents were 16 to 19 years old (*M_age_* = 17.07 years, *SD* = .86) and attended high school. Table 2 presents the demographic profile of the participating adolescents.

**Table 2:** Demographic Characteristics of the Participants by Country

Characteristic	Country					
	Sweden (*N* = 279)		Italy (*N* = 301)		Greece (*N* = 225)	
	*n*	%	*n*	%	*n*	%
Type of school						
Theoretical^a^	117	53.9%	110	39.6%	137	66.2%
Caring^b^	56	25.8%	0	0	0	0
Technical^c^	44	20.3%	168	60.4%	70	33.8%
SES						
Low	44	20.6%	18	7.2%	35	17.0%
Medium	78	36.4%	172	68.8%	47	22.8%
High	92	43.0%	60	24.0%	124	60.2%
Age (years)	*M*	*SD*	*M*	*SD*	*M*	*SD*
	17.06	.69	16.98	.87	17.22	.98

^a^Learning-goal centred school prepares students to enter the university. ^b^Social goal centred school strengthens students’ social skills. ^c^Learning-goal centred school prepares students to enter technical schools.

### Research Instruments

#### Socio-demographic Characteristics

Participants were given a socio-demographic questionnaire that ascertained information about participants’ gender, age, nationality, school, and fathers’ and mothers’ educational qualification. This last indicator was used to compute SES, a three-level variable: low (when both parents attended only elementary and/or middle school, or when one of them attended elementary/or middle school and the other one high school), medium (when both parents attended high school, or when one of them attended elementary/or middle school and the other one university) and high (when both parents attended university, or when one of them attended high school and the other one university).

#### Parenting Styles and Dimensions Questionnaire

The retrospective version of the PSDQ (Robinson, Mandleco, Olsen, & Hart, 1995, 2001; Tagliabue, Olivari, Bacchini, Affuso, & Confalonieri, 2014) was translated and adapted in the language of the country where it was administered by the researchers. Each country followed the same translation procedure. First, independent translations were made and then the PSDQ was back translated by two English-speaking psychologists. Adolescents were told, “You will find, described below, a list of behaviors that parents show with their children. Please, think back and recall your childhood, and your relationship with your parents, and indicate how much both your father and your mother behaved in that manner with you.” The PSDQ consists of 62 items assessing the three parenting styles suggested by Baumrind (1967, 1971): the authoritative, the authoritarian and the permissive. Twenty-seven items belonged to the authoritative style (e.g., “My parents encouraged me to talk about my troubles,” Cronbach’s Alpha ranged .91-.96), 20 items to the authoritarian style (e.g., “My parents slapped me when I misbehaved,” Cronbach’s Alpha ranged .83-.87), and 15 items to the permissive style (e.g., “My parents stated punishments to me but didn’t actually do them,” Cronbach’s Alpha ranged .50-.71). Two versions were evaluated, the first regarding the father’s parenting style and the second, the mother’s parenting style. Adolescents were asked to answer using a 5-point scale anchored by 1 (never) and 5 (always). For further information on PSDQ reliability and validity, see the review of Olivari et al. (2013).

### Procedure

The participants were recruited from high schools in Greece, Italy and Sweden. The purpose of the study was explained to the headmaster and class teachers, who gave their permission. Moreover, in Italy and Greece, parents gave written consent for their minor children, whereas in Sweden the parents do not have to give their consent when the adolescent is over 15 years old. Approval to conduct the study in Italy and in Sweden was obtained, whereas such approval was not necessary in Greece. The participating students were first informed of the purpose and usefulness of the study and the importance of their participation, and they were given appropriate instructions for completing the questionnaires in their classrooms. Students completed the anonymous questionnaire during the school lessons in the presence of researchers. The completion of questionnaires took approximately 45 minutes.

## Results

Three mixed 2(parent; mother and father)*2(gender; girl and boy)*3(countries; Sweden, Italy and Greece)*3(SES; low, medium, high) ANOVAs were conducted separately for each parenting style. Only principal effects were tested in the model, due to the low number of cases within the interaction cells.

Regarding the authoritative style, Parent (*F*(1, 664) = 137.66, *p* < .001; η^2^ = .17), Country (*F*(2, 664) = 7.42, *p* < .01; η^2^ = .02), and SES (*F*(2, 664) = 5.60, *p* < .01; η^2^ = .02) principal effects were significant. Regarding the authoritarian style, Parent (*F*(1, 664) = 8.18, *p* < .01; η^2^ = .01), Country (*F*(2, 664) = 19.60, *p* < .001; η^2^ = .06), and Gender (*F*(1, 664) = 6.20, *p* < .05; η^2^ = .01) principal effects were significant. Regarding the permissive style, Parent (*F*(1, 664) = 6.66, *p* < .05; η^2^ = .01), Country (*F*(2, 664) = 70.25, *p* < .001; η^2^ = .17), and Gender (*F*(1, 664) = 5.80, *p* < .05; η^2^ = .01) principal effects were significant.

In order to verify the interaction effects, we tested one mixed 2(parent; mother and father) *3(countries; Sweden, Italy and Greece)*3(SES; low, medium, high) ANOVA on authoritative style, considering only the independent variables which were significant in the previous model (see Tables 3 and 4). Regarding authoritarian and permissive styles, we tested a mixed 2(parent; mother and father)*2(gender; girl and boy)*3(countries; Sweden, Italy and Greece) ANOVA (see Tables 5 and 6).

**Table 3:** Principal and Interaction Effects for Authoritative Parenting Style

Effects	*F*	*df*	η^2^
Parent	122.17***	1, 661	.16
Country	2.46	2, 661	.01
SES	4.56*	2, 661	.01
Parent*Country	6,62**	2, 661	.02
Parent*SES	0,06	2, 661	
Country*SES	1.39	4, 661	.01
Parent*Country*SES	1.49	4, 661	.01

*p < .05. **p < .01. ***p < .001.

**Table 4:** Descriptive Statistics for Authoritative Parenting Style

*SES*	Country							
	Sweden		Italy		Greece		Total	
	*M*	*SD*	*M*	*SD*	*M*	*SD*	*M*	*SD*
Father								
Low	3.38	0.91	3.19	0.74	3.12	0.82	3.25	0.85
Medium	3.67	0.75	3.32	0.80	3.31	1.01	3.41	0.83
High	3.53	0.84	3.36	0.78	3.62	0.74	3.53	0.79
Total	3.55	0.83	3.32	0.79	3.46	0.84	3.44	0.82
Mother								
Low	3.66	0.83	3.48	0.61	3.56	0.66	3.59	0.73
Medium	3.76	0.71	3.66	0.64	3.84	0.72	3.71	0.68
High	3.77	0.79	3.68	0.60	3.98	0.54	3.85	0.66
Total	3.74	0.77	3.65	0.63	3.88	0.62	3.75	0.68

Regarding authoritative style, a significant principal effect was found for Parent (mothers were perceived as more authoritative than fathers) and SES, whereas significant interaction effect were found for Parent*Country (see Tables 3 and 4). Bonferroni post hoc analyses regarding the principal effect of SES revealed that parents with high SES were perceived as more authoritative than parents with low SES (mean difference = .27, *p* < .01). Moreover, despite the significance of Parent*Country interaction effect, paired *t*-test analyses conducted on Parent variable did not highlight differences with the principal effect, separately considering each of the three countries.

**Figure 1:**
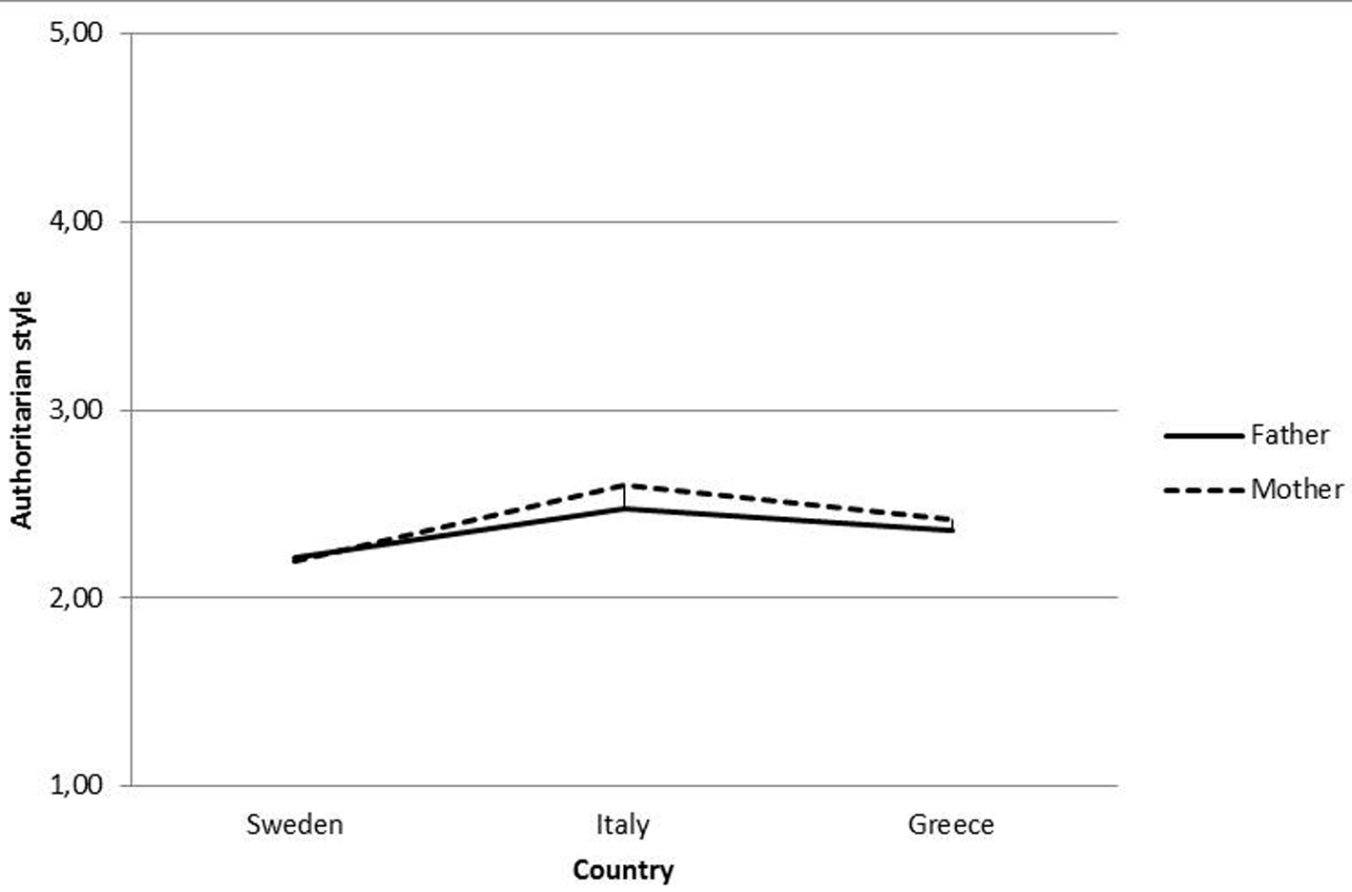
Interaction effect Parent*Country.

Regarding authoritarian style, a significant principal effect was found for Parent (mothers were perceived as more authoritarian than fathers), Gender and Country, whereas significant interaction effect was found for Parent*Country (see Tables 5, 6 and Figure 1).

**Table 5:** Principal and Interaction Effects for Authoritarian and Permissive Parenting Styles

Effects	Authoritarian			Permissive		
	*F*	*df*	η^2^	*F*	*df*	η^2^
Parent	7.45**	1, 696	.01	6.63*	1, 696	.01
Gender	6.46*	1, 696	.01	5.21*	1, 696	.01
Country	23.66***	2, 696	.06	72.97***	2, 696	.17
Parent* Gender	0.47	1, 696		0.01	1, 696	
Parent*Country	4.47*	2, 696	.01	2.35	2, 696	
Gender*Country	1.75	2, 696		.98	2, 696	
Parent*Gender*Country	.55	2, 696		3.01	2, 696	

*p < .05. **p < .01. ***p < .001.

**Table 6:** Descriptive Statistics for Authoritarian and Permissive Parenting Styles

Parenting Style	Country							
	Sweden		Italy		Greece		Total	
	*M*	*SD*	*M*	*SD*	*M*	*SD*	*M*	*SD*
Father								
Authoritarian								
Girl	2.15	0.49	2.39	0.61	2.36	0.64	2.30	0.59
Boy	2.30	0.56	2.58	0.64	2.36	0.56	2.43	0.60
Total	2.22	0.52	2.48	0.63	2.36	0.60	2.36	0.60
Permissive								
Girl	2.23	0.39	1.83	0.49	2.33	0.45	2.10	0.50
Boy	2.32	0.51	1.98	0.50	2.31	0.37	2.18	0.49
Total	2.27	0.45	1.90	0.50	2.32	0.41	2.14	0.50
Mother								
Authoritarian								
Girl	2.13	0.45	2.55	0.59	2.42	0.59	2.37	0.58
Boy	2.29	0.62	2.66	0.60	2.41	0.55	2.47	0.61
Total	2.20	0.54	2.60	0.59	2.41	0.57	2.42	0.59
Permissive								
Girl	2.27	0.42	1.86	0.46	2.35	0.41	2.13	0.49
Boy	2.31	0.61	1.98	0.52	2.42	0.38	2.21	0.54
Total	2.29	0.51	1.91	0.49	2.39	0.40	2.17	0.52

Independent *t*-test analysis showed that adolescent males perceived as more authoritarian their mothers (*t*(700) = -2.20, *p* < .05) and fathers (*t*(700) = -2.81, *p* < .01) than females. Bonferroni post hoc analyses regarding the principal effect of Country revealed that Swedish parents were perceived as significantly less authoritarian than Italian (mean difference = -.33, *p* < .001) and Greek parents (mean difference = -.18, *p* < .01) and that Greek parents were perceived as less authoritarian (mean difference = -.15, *p* < .01) than Italian parents. Paired *t*-test analyses conducted on Parent separately, considering each of the three countries, revealed that the only significant difference was found for Italy where fathers were perceived as less authoritarian than mothers (*t*(277) = -3.87, *p* < .001).

Regarding permissive style, only principal effects for Parent (mothers were perceived as more permissive than fathers), Gender and Country were found (see Tables 5 and 6). Independent *t*-test analysis showed that adolescent males perceived as more permissive their mothers (*t*(700) = -2.18, *p* < .05) and fathers (*t*(700) = -2.26, *p* < .05) than females. Bonferroni post hoc analyses regarding the principal effect of country revealed that Swedish (mean difference = .37, *p* < .001) and Greek parents (mean difference = .44, *p* < .001) were perceived as more permissive than Italian parents.

## Discussion

Little attention has been paid in literature to the investigation of the differences between parenting styles among Nordic and Mediterranean countries.

The present study attempted to explore differences and similarities in adolescents’ retrospective perceptions of authoritative, authoritarian and permissive parenting styles for their fathers and mothers in Sweden, Italy and Greece, considering the potential role played by the country legislation on family matters in affecting parenting styles across the three different countries. In particular, it examined the extent to which parental role, adolescent gender, country of origin and SES are related to these perceptions. Moreover, this study examines differences between maternal and paternal parenting styles, because frequently in the literature these styles are jointly studied or the focus is directed towards the mother, leaving in the background the figure of the father (Ge et al., 1996; Parke, 2000; Rohner & Britner, 2002; Sentse et al., 2009).

The descriptive statistics showed that in Sweden, Italy and Greece, the authoritative style, according to both boys’ and girls’ perceptions, is the most frequently adopted by both fathers and mothers. In all three countries, the legislations and policies concerning the family role in educating and caring for children stress the importance for parents to raise children by treating them with respect, supporting and protecting them, and prohibiting the use of physical punishment as a discipline method. At a psychological level, these government suggestions, together with other elements (i.e. personality, marital relations, work and social network) which are likely to influence parenting behaviours (Belsky, 1984; Bronfenbrenner, 1979, 1986), have found their operationalization in the parental use of a parenting style that can be described as democratic and respectful of the children’s needs and personal inclinations.

Another interesting finding is that adolescents perceived their mothers as being more authoritative, authoritarian and permissive than fathers. A possible explanation for this result could be that, despite the increasing involvement of fathers in the children’s education, the educational tasks are still perceived as more maternal than paternal by the adolescents.

Moreover, the results did not show any differences in perceptions about the authoritative style between boys and girls. However, boys perceived both their mothers and fathers as being more authoritarian and permissive than girls. These data are in line with previous results, suggesting that male adolescents, compared to females, reported higher scores for negative perceived parenting styles (Brand et al., 2011). These data may reflect parents’ difficulty in using democratic upbringing approaches with boys. Further studies should deepen the investigation of this aspect.

Regarding authoritarian parenting style, Swedish adolescents believe that both their parents adopt this style less frequently, when compared with Greek and Italian adolescents. The restricted use of authoritarian parenting approaches in Sweden may be linked with the long Swedish tradition of considering children as equal (Key, 1995) and as individuals that have to be supported and not directed (Hallden, 1991). Compared to Swedish and Greek participants, Italian adolescents perceived their parents as more authoritarian. Moreover, they believe that their fathers adopt a less authoritarian parenting style compared to their mothers. Fathers seem to have left their ethical tasks, moving on to the affective tasks that traditionally belong to the maternal figure (Bacchini et al., 2003). It is possible to hypothesize that now mothers are becoming more committed to authoritarian practices into fulfill the role that was once performed by fathers.

Finally, Swedish and Greek adolescents reported higher levels of permissive styles for both their fathers and mothers, when compared with their Italian peers. This finding is in accordance with other findings showing that Swedish parents have a more tolerant behaviour in regard to disobedience during childhood (Durrant et al., 1999), suggesting the use of a more permissive parenting style.

In the present work, we also considered the relation between SES and parenting styles. Consistent with previous studies (Bronfenbrenner, 1958; Chen et al., 1997; von der Lippe, 1999) parents with high educational levels were described as more authoritative than parents with low educational levels in Sweden, Italy and Greece. On the contrary, in our sample, parents with low educational levels were not perceived as more authoritarian than parents with high education levels.

This study has some limitations. First, these findings are based on a correlational study, so caution should be taken when inferring conclusions. Secondly, PSDQ provides the possibility of assessing both paternal and maternal authoritative, authoritarian and permissive parenting styles; however, permissive parenting style in Greece had a low Cronbach’s Alpha. Thirdly, in this study we measured SES by a single indicator, multiple indicators or composite indices may have provided more complete information. Further studies should consider the possibility of approaching this theme with a cross-informant method in order to assess the agreement between parents and adolescents across different countries.

In sum, the present study, which is an attempt to compare one Nordic country, Sweden, and two Mediterranean countries, Italy and Greece, provides an interesting contribution to parenting styles literature, showing how country legislation concerning family matters and SES could be related to parental behaviours (Belsky, 1984; Bronfenbrenner, 1979, 1986; Garbarino et al., 2002). Moreover, throughout the choice of assessing parenting styles with PSDQ (Robinson et al., 1995, 2001; Tagliabue et al., 2014), we had the chance to assess both maternal and paternal parenting styles, an aspect frequently neglected in literature.
